# Effects of antioxidant vitamins (A, D, E) and trace elements (Cu, Mn, Se, Zn) administration on gene expression, metabolic, antioxidants and immunological profiles during transition period in dromedary camels

**DOI:** 10.1186/s12917-024-03959-3

**Published:** 2024-03-13

**Authors:** Ahmed El-Sayed, Eman Ebissy, Ragab Mohamed, Ahmed Ateya

**Affiliations:** 1 Department of Animal Health and Poultry, Animal and Poultry Production Division, Desert Research Center (DRC), Cairo, Egypt; 2 Department of Theriogenology, Faculty of Veterinary Medicine, Aswan University, Aswan, Egypt; 3 Department of Development of Animal Wealth, Faculty of Veterinary Medicine, Mansoura University, Mansoura, Egypt

**Keywords:** Camel, Transition period, Antioxidants, Traces elements, Gene expression, Immunity

## Abstract

**Background:**

Nutrition has a primary role for optimum expression of genetic potential, and most of the farmers have limited resources of green fodder. Hence, a fat-soluble vitamin, especially vitamin A and E and trace elements remained most critical in the animal’s ration and affects their productive and reproductive performance adversely. Animals cannot be able to produce these vitamins in their bodies; hence, an exogenous regular supply is needed to fulfil the physiological needs and to maintain high production performance. This study elucidated effects of antioxidant vitamins (A, D, E) and trace elements (Cu, Mn, Se, Zn) administration on gene expression, metabolic, antioxidants and immunological parameters in dromedary camels during transition period.

**Results:**

At 0 day, there were no appreciable differences in the expression patterns of the metabolic (*IGF-I, ACACA, SCD, FASN, LPL*, and *BTN1A1*) genes between the control and treatment groups, despite lower levels. A substantial variation in the mRNA levels of *SOD1, SOD3, PRDX2, PRDX3, PRDX4, PRDX6*, and *AhpC/TSA* was observed between the control and treatment groups, according to the antioxidant markers. In comparison to the control group, the treatment group displayed a significant up-regulation at 0 and 21 days. The treatment and control groups exhibited substantial differences in the mRNA values of *IL-1α, IL-1β, IL-6*, and *TNFα*, as indicated by immunological markers. In comparison to the control group, there was a noticeable down-regulation in the treatment group at 0 and + 21 days. But *IL10* produced the opposite pattern. No significant difference was observed in glucose, cholesterol, triglyceride, HDL, total protein, NEFA, BHBA, cortisol and IGF-1 levels between control and treatment group. The activity of serum GPx, SOD and TAC was significantly affected by time and treatment x time in supplemented groups as compared with control group. IL-1, IL-1, IL-6, and TNF were noticeably greater in the control group and lower in the treatment group. Additionally, in all groups, the concentration of all pro-inflammatory cytokines peaked on the day of delivery and its lowest levels showed on day 21 following calving. The IL-10 level was at its peak 21 days prior to calving and was lowest on calving day.

**Conclusion:**

The results demonstrated a beneficial effect of antioxidant vitamins and trace elements on the metabolic, antioxidant and immunological markers in dromedary camels throughout their transition period.

## Introduction

*Camelus dromedarius*, often known as the dromedary one-humped camel, was significant because it served as a multifunctional animal that could be used for transport and work as well as to produce meat, milk, leather, and other byproducts [1, 2]. In a variety of dry, semi-arid, and tropical regions of Asia, Africa, and Australia, they were well suited to the harsh environment and extreme temperatures [3, 4]. Most camels in these areas are typically raised on natural pastures settings with varying diets in terms of both quality and quantity. However, under these circumstances, the dietary needs of grazing animals, particularly those for micronutrients, are frequently not met, at least part of the year. For instance, grazing animals are more likely to experience deficiencies in one or more trace minerals, such as zinc (Zn), selenium (Se), copper (Cu), and cobalt (Co), which can result in slower growth and productivity as well as a higher mortality rate and increased susceptibility to a variety of diseases [5].

During the transition periods, from late gestation to early lactation, animals are likely to experience changes in their metabolic and endocrine conditions [6]. Homeorhetic alterations take place during this time to support lactogenesis and deliver nutrition to the developing foetus [7]. This increase in nutritional demand and decrease in food intake during this time contribute to the development of a negative energy balance (NEB), which if prolonged can lead to an immunosuppressive state that increases the risk of developing lipomobilization, ketosis, and hypocalcemia [8, 9].

The main cause of immunosuppression and increased disease vulnerability in transition dromedary camels is oxidative stress [10]. Reactive oxygen species (ROS) and lipid peroxidation are known to cause cellular damage to tissues, particularly immune cells, and are produced more often as a result of metabolic demands associated with late pregnancy, delivery, and the start of lactation [11]. Oxidative stress induced by free radicals can be prevented by repair, physical, and antioxidant defences [12].

Vitamins and minerals (macro and microelements) have a significant impact on dairy animals' physiological activity appropriate growth, production, and reproduction, immunity, oxidative metabolism, nutrition, and energy metabolism [13]. Significant pathologies appear in the organism as a result of deficiencies [14]. According to [15], vitamin A is a lipophilic molecule that is necessary for immune system function, growth, and differentiation of epithelial tissues, as well as for good health and fertility. Vitamin D is involved in bone formation, calcium balance, and other physiological processes critical to maintain dairy animals’ health and sustain productions. It also contributes to reproductive performance and mammary development [16]. Vitamin E is a crucial lipid-soluble antioxidant that can reduce hydrogen peroxide formation and prevent oxidative damage to the sensitive membrane lipids, thereby reducing oxidative stress and maintaining the integrity of cell membranes [17]. Vitamin E can also prevent peroxidation in the susceptible subcellular membrane [18]. Selenium (Se) acts as antioxidant factor because it is a crucial component of the glutathione peroxidase enzymes, which may eliminate lipid hydroperoxides and hydrogen peroxide (H_2_O_2_) [19]. Copper (Cu) and zinc (Zn) are necessary building blocks for a number of enzymes, including the oxidation–reduction reaction and Cu–Zn superoxide dismutase (SOD) [20]. According to [21], manganese (Mn) is an essential co-factor in enzymatic processes connected to metabolic control of gene expression. Additionally, there is strong evidence that these trace minerals, all contribute to reducing the harmful consequences of oxidative stress [13].

Variations in the gene expression of a variety of regulatory enzymes involved in the intermediate metabolism, according to [22], can be useful tools for enhancing genetic selection for the adaptation of livestock to difficult environmental conditions. One aspect of metabolic regulation is the transcriptional control of gene networks, which are collections of DNA segments that interact with transcription factors or nuclear receptors to control the concentration of key enzymes in cells. These "global" interactions might regulate the rate at which the genes in the network are translated into mRNA. Research on the entire genome, sub-networks, or potential genes at the mRNA level are all included in the large field of genomics [23].

Little information on the metabolic, antioxidant, and gene expression patterns in dromedary camels during the transition phase is available in the literature [24, 25]. Based on the benefits of vitamins and trace minerals on physiological processes, energy metabolism, and immune status, we hypothesized that supplementation with injectable vitamins and trace minerals would lessen the negative effects of mineral deficiencies on homeostasis, immune function, and disease development. Therefore, the goal of the current study was to assess how different exogenous vitamins (A, D, and E) and trace elements (Cu, Mn, Se, and Zn) affected gene expression, metabolic profiles, antioxidant and immunological characteristics in dromedary camels during the transition period.

## Materials and methods

### Animals

A total of thirty apparently healthy pregnant female dromedary camels, with a mean body weight of 512 kg (range: 390—634 kg) and a mean age of 20 years (range: 18 -22), were used in this study. Animals were obtained from Mariut Research Station, Desert Research Center, El-Amria, Alexandria, Egypt. Camels were housed in an open yard and fed on a maintenance ration composed of a concentrate mixture including 50% corn, 47% barley, 2% minerals, 1% salt, and Egyptian clover. Concentrate mixture was administered at a rate of 3 kg/head/day, while Egyptian clover hay (Trifolium alexandrinum) and fresh water were offered *ad libitium*. Pregnancies were all consequences of natural mating and detections were confirmed by ultrasound (Samsung Medison SONOACE R3 ultrasound system, South Korea). Camels were considered clinically sound on the basis of physical examination of heart, lungs, rumen and intestine and other vital signs in tandem with the preliminary findings of hematological examination [26].

### Study design

Three weeks prior to the expected time of calving, the investigated camels were randomly allocated into two equal-sized groups (15 camels each), and were assigned to receive one of the following treatments: a placebo which received twenty ml sterile saline solution subcutaneously (0.9% NaCl) and served as control group (G1)**;** ten ml of mineral solution, containing 5 mg of copper, 5 mg of selenium, 10 mg of manganese, 40 mg of zinc per ml (Minarin, Plexo pharm, Egypt 50 ml, s/c) and 10 ml of vitamin solution, containing 80,000 IU vitamin A, 40 000 IU vitamin D3, 20 mg vitamin E per ml, i.m.) were administered intramuscularly to each camel in the treatment group (G2).

### Clinical examination

The clinical examinations included measurements of heart and respiratory rates and recording of rectal temperatures as well as rumen movements was done as described by [27–30] at days -21, 0 and + 21 post-partum in both groups.

### Blood sampling

Ten millilitres of blood were collected from each camel via jugular vein puncture at the following time points: at the time of administration (-21 day), 0 (at time of calving), and + 21 (three weeks following the date of delivery). The samples were collected into vacutainer tube containing anticoagulant (EDTA or sodium fluoride) and without anticoagulant to yield whole blood or serum, respectively. The EDTA blood was used for real-time PCR assay while those in plain tubes were kept overnight at room temperature and centrifuged at 3000 rpm for 15 min. Only clear sera were collected then aliquoted and kept frozen at -20 ^0^C for subsequent biochemical analyses of energetic and oxidative stress markers.

### RNA extraction, reverse transcription and real time PCR

To extract total RNA from freshly drawn blood samples, Direct-zolTM RNA MiniPrep extraction kit was used following the manufacturer's guidelines. A Nanodrop (UV–Vis spectrophotometer Q5000/USA) was used to measure the quantity and purity of the isolated RNA. Using SYBR Green PCR Master Mix (2 × SensiFastTM SYBR, Bioline), the mRNA levels of metabolic, antioxidant, and immune indicators were measured. The primer sequences were designed according to the PubMed published sequence of *Camelus dromedaries* as shown in Table 1. The housekeeping gene *GAPDH* was used as an internal control. The PCR cycling conditions were 94 °C for 10 min followed by 40 cycles of 94 °C for 15 s, annealing temperatures as shown in Table 1 for 30 s and 72 °C for 30 s. The relative expression of each gene/sample compared to *GAPDH* gene was carried out and calculated according to the 2^−ΔΔCt^ method [31].

**Table 1:** Oligonucleotide primers sequence, annealing temperature and PCR product size of the studied genes

Gene	Primer	Product length (bp)	Annealing Temperature (°C)	Accession number	Source
*IGF-I*	F:5-CTTCCGGAGCTGTGATCTGA-3 R:5-TGTACTTCCTTCTGAGCCTTGG-3	123	59	MZ491851.1	Current study
*ACACA*	F:5- TTCCAATGTCTGCTTGTCCGT-3 R:5- GCTGAGCAGAGTCGAAGAACA-3	190	60	KP236453.1	Current study
*SCD*	F:5- ATGGTTGAGCCCCAGTGATG-3 R:5- TCCAGCCTTGCGTAGAGTTG-3	150	60	XM_010971047.2	Current study
*FASN*	F:5- GGTGGACTCGCTGAAGAACA-3 R:5- ATACCAGGACGCACCGAATC-3	149	62	XM_010950631.2	Current study
*BTN1A1*	F:5- ACGTCCTACACACGTTCACC-3 R:5- CCAAAACCTCTCTCGGCGAT-3	154	60	XM_010980175.2	Current study
*SOD 3*	F:5-GACACCTCTCCAAAAGCCCA-3 R:5-GCACATGGTTGGAGGCCTTA-3	169	60	XM_031436563.1	Current study
*CAT*	F:5-GATGAGAAGCCGAAGAACGC-3 R:5-ATGCTTGGCCTCATAGGCAG-3	136	60	XM_011000575.2	Current study
*GPX*	F:5-CACCTGGTCTCCAGTATGCC-3 R:5-TCGATGTCAGGCTCGATGTC-3	129	59	XM_031470314.1	Current study
*PRDX2*	F:5-CCAAACACAACTAGGCTGGC-3 R:5-CCTTTAGGGCCATGGACTGT-3	137	58	XM_010993804.2	Current study
*GAPDH*	F:5-TCGATCCCCCAACACACTTG-3 R:5-TGATGGTGCATGACAAGGCA-3	106	58	XM_010990867.2	Current study

*IGF-I* Insulin-like growth factor-1, *ACACA *Acetyl-*CoA* carboxylase, *SCD *Stearoyl-*CoA* desaturase, *FASN* Fatty acid synthase, *BTA1N1* Butyrophilin Subfamily 1 Member *A1*, *SOD3* superoxide dismutase 3, *CAT*  catalase, *GPX* glutathione peroxidase and *PRDX2 *peroxiredoxin 2

### Biochemical profile

The following kits were used to quantify serum concentration of total protein, glucose and cholesterol (Gamma Trade Company, Egypt); beta- hydroxylbutyrate, (Cayman chemical, USA; Item No: 700190); NEFA (Randox laboratories Ltd., Crumlin Co., Antrim, UK); HDL and triglyceride (Spinreact Company, Spain); Total antioxidant capacity (TAC), glutathione peroxidase (GPx), super oxide dismutase (SOD) (Biodiagnostic Egypt); Cortisol, ParameterTM USA were used (Ref: KGE008B and KGE014); IGF1 by DRG test kits (Germany), IL-1 α, IL-1β, IL-6, TNF-α and IL-10 by RayBiotech companyVR ELISA kits.

### Statistical analysis

- *H*_*0*_: Antioxidant vitamins (A, D, E) and trace elements (Cu, Mn, Se, Zn) could not modulate on gene expression pattern, metabolic and reproductive profiles during transition period in dromedary camels.
- *H*_*A*_: Antioxidant vitamins (A, D, E) and trace elements (Cu, Mn, Se, Zn) could modulate on gene expression pattern, metabolic and reproductive profiles during transition period in dromedary camels.

The data was analysed by using the SPSS software system (version 22). All the data obtained from the study were expressed as mean ± standard error and were analyzed by two-way ANOVA. Tukey"s multiple comparison test was used to find significance at the 5 percent level. The effect of treatment group (Gr), days (D) of the periparturient period, and their interactions (Gr × D) were estimated using the statistical model shown below

$${\varvec{Y}}{\varvec{i}}{\varvec{j}}{\varvec{k}}\boldsymbol{ }=\boldsymbol{ }{\varvec{\mu}}\boldsymbol{ }+\boldsymbol{ }{\varvec{G}}{\varvec{r}}{\varvec{i}}\boldsymbol{ }+\boldsymbol{ }{\varvec{D}}{\varvec{j}}\boldsymbol{ }+\boldsymbol{ }({\varvec{G}}{\varvec{r}}{\varvec{D}}){\varvec{i}}{\varvec{j}}\boldsymbol{ }+\boldsymbol{ }{\varvec{e}}{\varvec{i}}{\varvec{j}}{\varvec{k}}$$

where, **Yijk** is a dependent variable,

**μ** is the overall mean of the population,

**Gri** is the effect of micronutrients feeding (i = 4),

**Dj** is the effect due to the measurement days (j = 4—7 based on parameters studied),

**(GrD)ij** is the effect due to treatment group by measurement days’ interactions,

and **eijk** is the residual error.

## Results

### Clinical findings

The prepartum injections of antioxidant vitamins (A, D, E) and trace elements (Cu, Mn, Se, Zn) had no significant effect on temperature, pulse, respirations and rumen motility between control and treatment group at different time points of the study (Table 2).

**Table 2:** Mean values (M ± SD) of temperature, pulse, respiration and rumen movements in control (*n* = 15) and treatment group (*n* = 15) she-camels during the transition period

Variables	Control			Treatment			Reference values
	-21 day	0 day	+ 21 day	-21 day	0 day	+ 21 day	
Temperature (°C)	37.8 ± .0.3^a^	37.5 ± .0.31^a^	37.6 ± 0.29^a^	38 ± 0.28^a^	37.9 ± 0.35^a^	38.1 ± 0.21^a^	(37.2 ± 0.77) [32] or (37.52 ± 0.09) [29]
Pulse (Beats/min)	33.3 ± 2.5^a^	32.5 ± 2.6^a^	33 ± 2.4 ^a^	29.6 ± 1.9^a^	31.2 ± 1.89^a^	32.4 ± 2.1^a^	32–36) [27] or (24–48/min) [33]
Respiration (Breaths/min)	10.8 ± 1.2^a^	10.7 ± 1.3^a^	11.1 ± 1.21^a^	11.9 ± 1.8^a^	12.1 ± 1.7^a^	12.2 ± 1.9^a^	(12.55 ± 0.30) [29] or (8–18) [34]
Rumen motility (Movements/2 min)	3.2 ± 0.6^a^	3.4 ± 0.8^a^	3.5 ± 0.5^a^	3.3 ± 0.0.3^a^	3.4 ± 0.33^a^	3.5 ± 0.29^a^	(4.25 ± 0.14) [29] or (4.3 ± 0.14) [35]

^a^Means superscripts a in the same raw are not significantly different (*P* < 0.05)

### Effects of vitamins (A, D, E) and trace elements (Cu, Mn, Se, Zn) on gene expression of metabolic, antioxidant and immunological markers during transition period

**Fig. 1:**
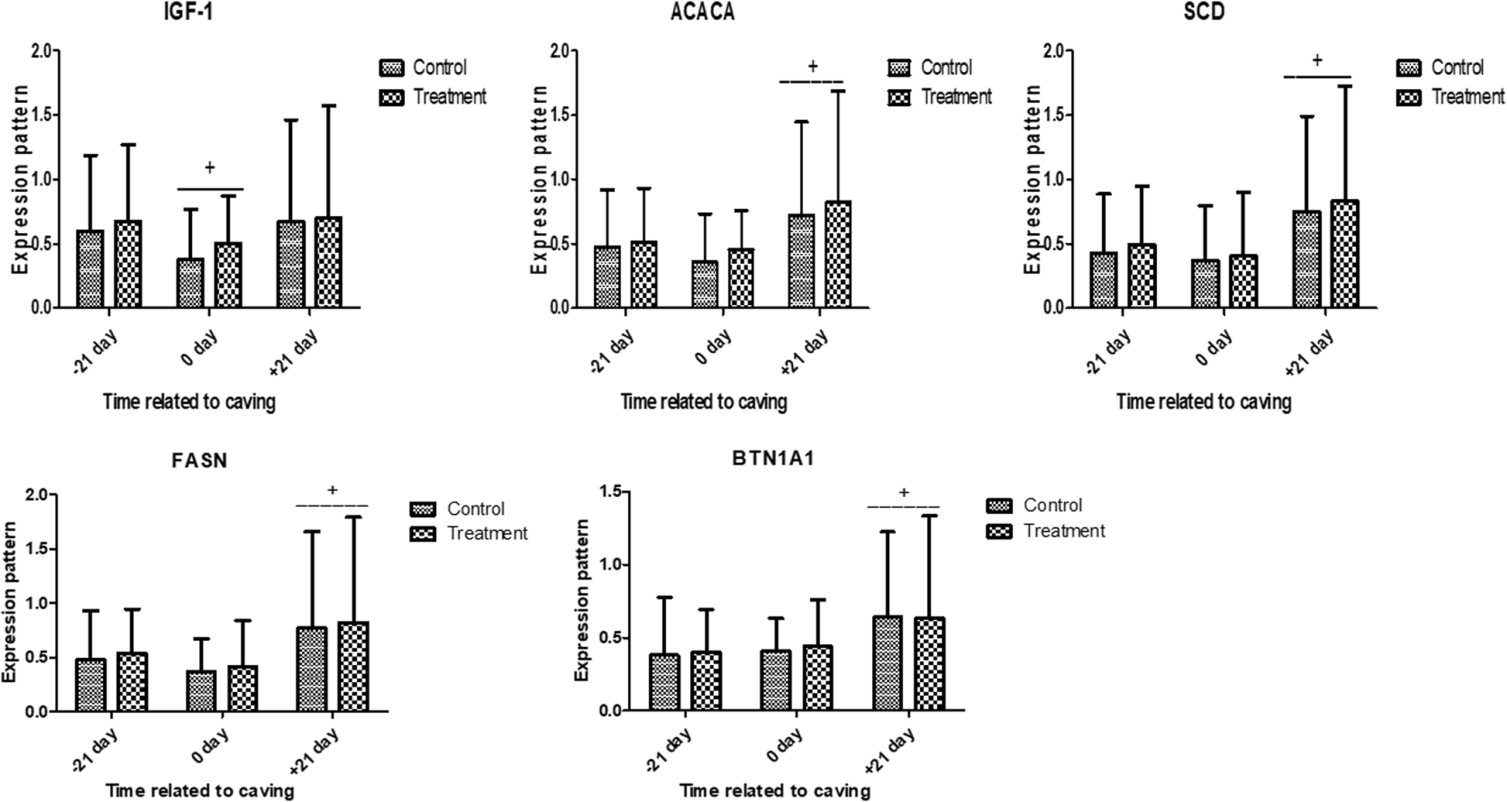
Relative expression patterns of metabolic genes in control and antioxidant vitamins (A, D, E) and trace elements (Cu, Mn, Se, Zn) treated dromedary camels (Mean ± SD). The superscript (*) indicates a significant difference within the groups, while the superscript ( +) indicates a significant difference over time

**Fig. 2:**
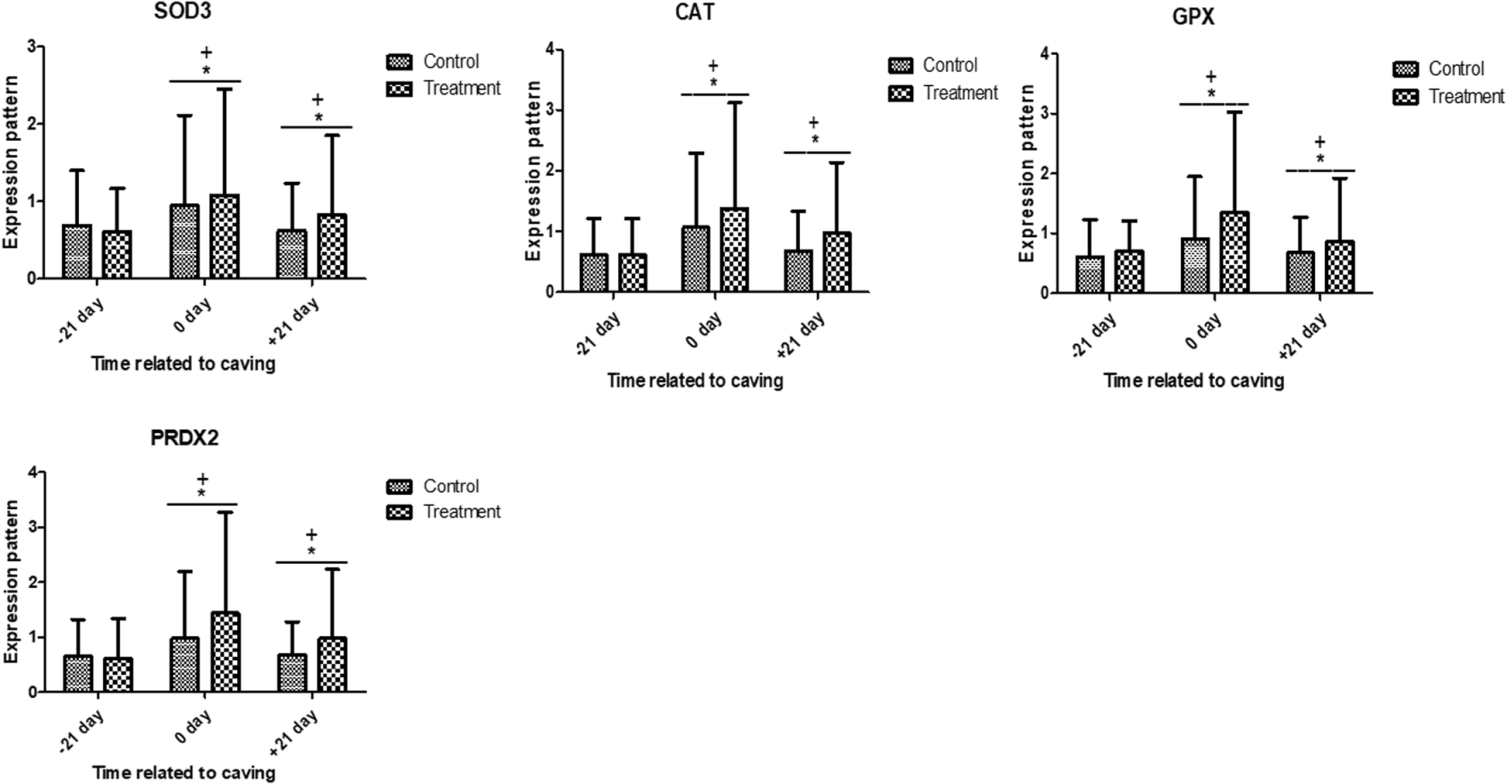
Relative expression patterns of antioxidant genes in control and antioxidant vitamins (A, D, E) and trace elements (Cu, Mn, Se, Zn) treated dromedary camels (Mean ± SD). The superscript (*) indicates a significant difference within the groups, while the superscript ( +) indicates a significant difference over time

**Fig. 3:**
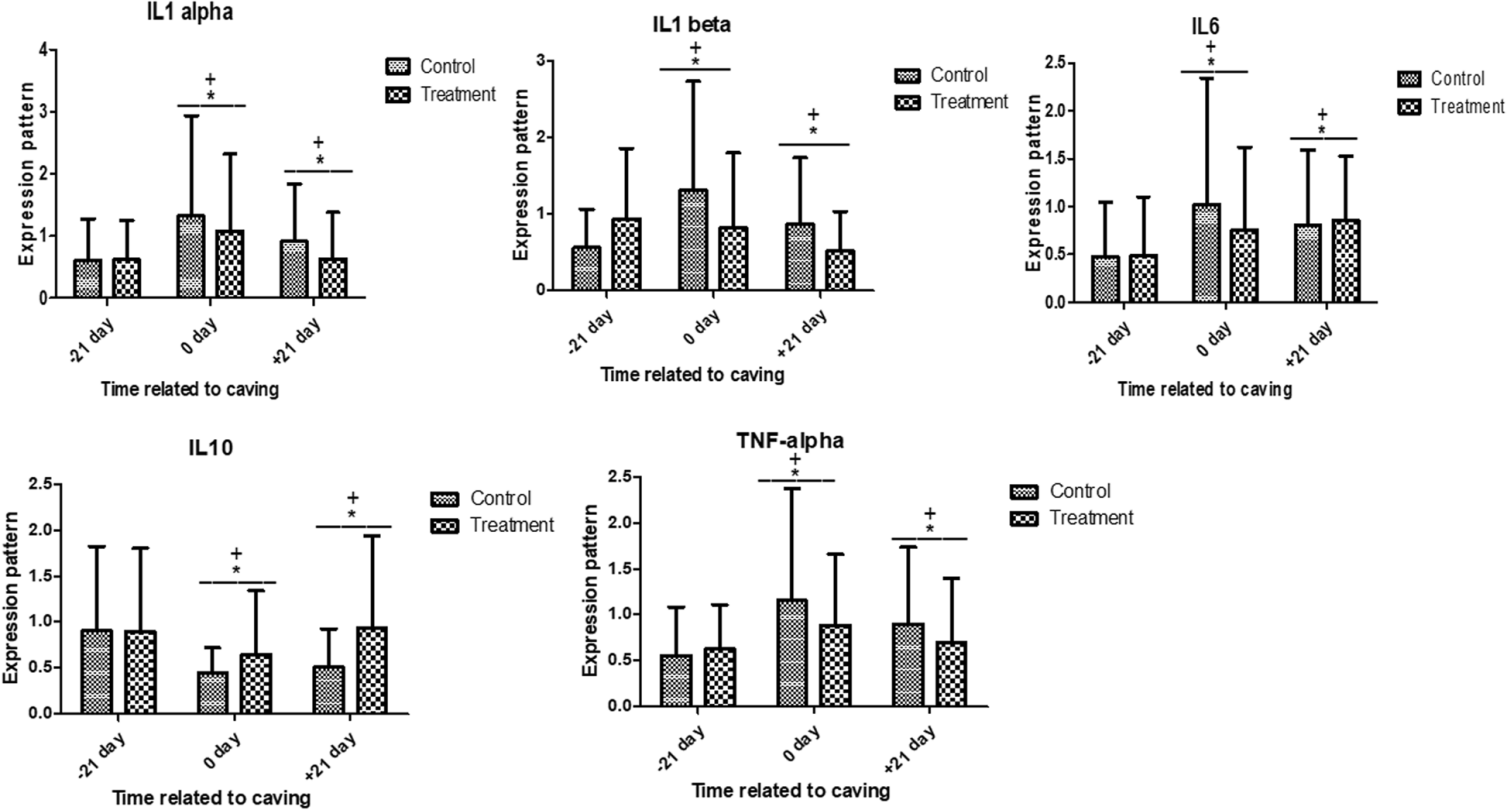
Relative expression patterns of immune genes in control and antioxidant vitamins (A, D, E) and trace elements (Cu, Mn, Se, Zn) treated dromedary camels (Mean ± SD). The superscript (*) indicates a significant difference within the groups, while the superscript ( +) indicates a significant difference over time

Results of the expression pattern of metabolic (*IGF-I, ACACA, SCD, FASN,* and *BTN1A1*), antioxidant (*SOD3, CAT, GPX,* and* PRDX2*, and immune (*IL-1α, IL-1β, IL-6, IL10*, and *TNFα*) genes in the control and treated groups during transition period are depicted in Figs. 1, 2 and 3; respectively. There were no significant alterations in the expression profile of metabolic genes between control and treatment group with lower values at 0 day in both groups (Fig. 1).

The gene expression of antioxidant markers is shown in Fig. 2, there was significant mRNA level of *SOD3, CAT, GPX,* and* PRDX2* markers between control and treatment groups with the significant highest transcript level in 0, and + 21 days than their values in -21 day. The treated group exhibited a significant up-regulation than control group at 0, and 21 days.

With regard to immune indicators, there was a notable difference in the mRNA values between the treatment and control groups, with both groups showing a significant up-regulation at 0 and + 21 days compared to -21 days (Fig. 3). At 0 and + 21 days, the treatment group showed a discernible down-regulation of *IL-1α, IL-1β, IL-6*, and *TNFα* compared to the control group. However, *IL10* resulted in the opposite pattern.

### Effects of vitamins (A, D, E) and trace elements (Cu, Mn, Se, Zn) on metabolic, antioxidant and immunological profile during transition period

**Fig. 4:**
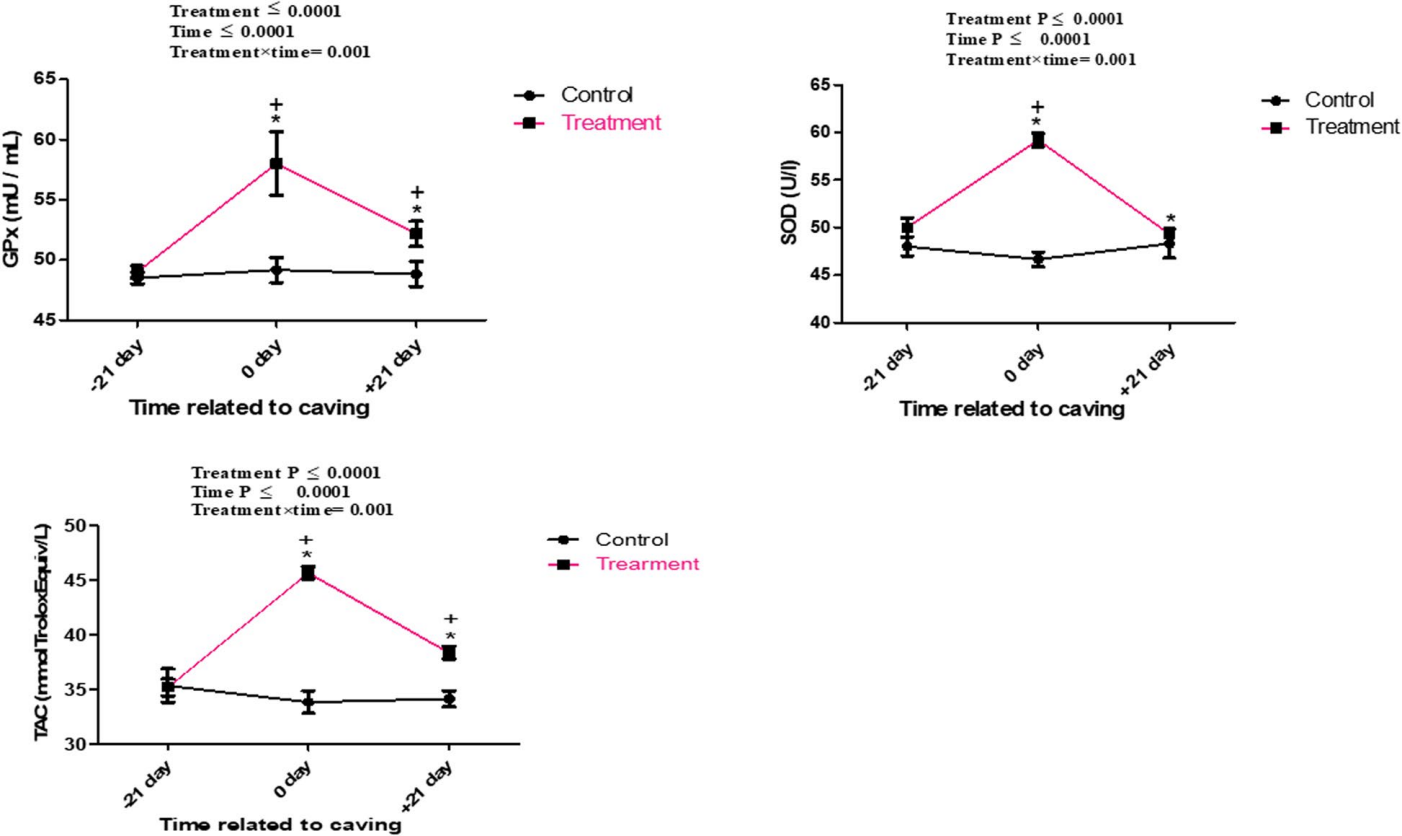
Time course of Serum GPx (Mu/ml), SOD (Mu/ml), TAC (mmolTroloxEquiv/L) concentrations in control and treatment groups of she camel (Mean ± SD). The superscript (*) indicates a significant difference within the groups, while the superscript ( +) indicates a significant difference over time

**Fig. 5:**
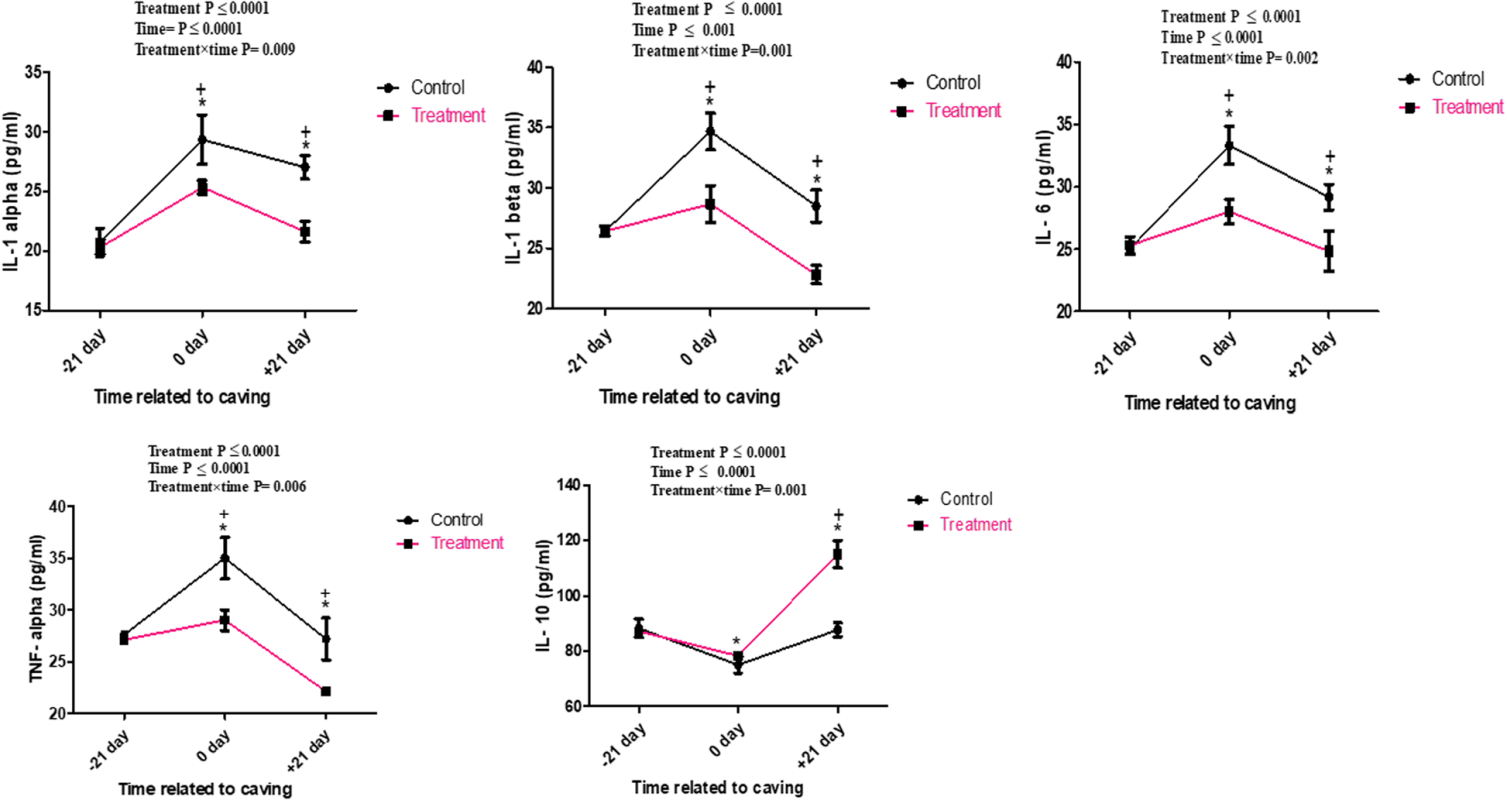
Time course of Serum of pro-inflammatory cytokines (pg/ml) including IL-1α, IL-1β, IL-6, TNFα and IL-10 in control and treatment groups of she camel. The superscript (*) indicates a significant difference within the groups, while the superscript ( +) indicates a significant difference over time

Biochemically, blood glucose, cholesterol, triglyceride, HDL, total protein, NEFA, BHBA, cortisol and IGF-1 levels were not significantly affected by treatment (*P* ˃ 0.001) and by treatment x time (*P* ˃0.05) but significantly affected by time (*P* < 0.05) between the examined groups. The higher values of glucose, NEFA, cortisol and lower levels cholesterol, triglyceride, total protein and IGF-1 were observed on the parturition day in treatment and control groups ( < 0.0001). However, higher BHBA and HDL values were detected in the third week postpartum (*P* = 0.02 and *P* < 0.0001), respectively in both groups (Table 3). The serum concentrations of GPx, TAC and SOD are displayed in Fig. 4. The GPx, and TAC was significantly affected by time (*P* = 0.0001) and treatment x time (*P* = 0.001) in supplemented groups at calving day and 21 day after parturition compared with those of control. The maximal concentration of serum GPx and TAC was observed in treatment group at calving day. The activity of serum SOD, was significantly affected by time (*P* < 0.0001) and treatment x time (*P* < 0.0001) in supplemented groups at calving day compared with those of control. The maximal concentration of serum SOD was observed in treatment group at calving day. The serum concentrations of pro-inflammatory (IL-1α, IL-1β, IL-6, and TNF-α) and anti-inflammatory (IL-10) cytokines are showed in Fig. 5. Pro-inflammatory cytokine concentrations (IL-1α, IL-1β, IL-6, and TNF-α) were noticeably greater in the control group and lower in the treatment group. Pro-inflammatory cytokines were significantly affected by time (*P* < 0.05) and treatment x time (*P* < 0.05) in supplemented groups at calving day and 21 day after parturition compared with those of control. The anti-inflammatory cytokine value (IL-10) was much lower in the control group as compared with treatment. The serum level of IL-10 was significantly affected by time (*P* < 0.05) and treatment x time (*P* < 0.05) in supplemented groups as compared with control. The highest concentration of serum IL-10 was observed in treatment group at 21 day after parturition.

**Table 3:** Means of minimum squares ± standard error of the mean (SE) and *P* value for biochemical parameters in control (*n* = 15) and treatment she camel (*n* = 15)

Variable	Group	Mean ± SE	*P*_*Treatment*_	*P*_*Day*_	*P*_Treatment*Day_
Glucose (mg/dl)	Control	118.5 ± 0.96	0.36	< 0.0001	0.61
	Treatment	119.7 ± 0.96			
Cholesterol (mg/dl)	Control	121.7 ± 1.2	0.24	0.001	0.46
	Treatment	123.9 ± 1.2			
Triglyceride (mg/dl)	Control	55.3 ± 0.6	0.89	< 0.0001	0.29
	Treatment	55.4 ± 0.6			
HDL (mg/dl)	Control	0.22 ± 0.49	0.69	< 0.0001	0.92
	Treatment	0.23 ± 0.49			
Total protein (g/dl)	Control	5.1 ± 0.03	0.89	< 0.0001	0.85
	Treatment	5.2 ± 0.03			
NEFA (mmol/L)	Control	4.167 ± 0.06	0.40	< 0.0001	0.16
	Treatment	3.943 ± 0.06			
BHBA (mmol/L)	Control	0.97 ± 0.04	0.27	< 0.0001	0.70
	Treatment	0.91 ± 0.04			
Cortisol (ug/dl)	Control	121.7 ± 1.2	0.89	< 0.0001	0.57
	Treatment	123.9 ± 1.2			
IGF1 (ng/ml)	Control	49.6 ± 0.2	0.09	< 0.0001	0.13
	Treatment	50.1 ± 0.2			

*HDL* High-density lipoprotein, *BHBA* beta-hydroxybutyrate, *NEFA *non-esterified fatty acids, *IGF-1* insulin-like growth factor-1

## Discussion

Compared to dairy cattle, dromedary camels' transition period has received very less attention, despite the existence of a few reports on camels (*Camelus bactrianus)* [25]; guanacos (*Lama guanicoe*); and llamas (*Lama glama*) [11]. Regrettably, no published data investigating the effect of antioxidant vitamins (A, D, and E) and trace elements (Cu, Mn, Se, and Zn) existed on certain metabolic, antioxidant, immunological and gene expression patterns in dromedary camels during transition period in Egypt.

Prepartum injections of antioxidant vitamins (A, D, E) and trace elements (Cu, Mn, Se, Zn) did not significantly affect body temperature, pulse, respirations and rumen motility between studied groups at different time points. Values of temperature, pulse and respiration were within the reference ranges reported by to [27, 29, 32–35]. All camels demonstrated normal laboring and delivered a single calf without obvious clinical illness. These findings were in part similar to that given by [36].

In this context, the expression profiles of metabolic (*IGF-I, ACACA, SCD, FASN,* and *BTN1A1*) genes in the control and treatment groups showed decreased values at 0 day, but no discernible changes. In terms of antioxidant marker gene expression, there was a significant difference in the mRNA levels of *SOD3, CAT, GPX,* and* PRDX2* between the control and treatment groups. The transcript levels were highest in 0 and + 21 days compared to -21 days. At 0 and + 21 days, the treatment group showed a considerable up-regulation compared to the control group. Regarding immunological markers, there was a notable difference in the mRNA values of *IL-1α, IL-1β, IL-6,* and *TNFα* between the treatment and control groups, with both groups showing a significant up-regulation at 0 and + 21 days compared to -21 days. At 0 and + 21 days, there was a discernible down-regulation in the treatment group compared to the control group. However, *IL10* evoked an opposite trend. Based on alterations in the expression pattern of immune and antioxidant genes, we could postulate the enhanced antioxidant and immune effects as a result of supplementation of antioxidant vitamins (A, D, E) and trace elements (Cu, Mn, Se, Zn).

To the best of our knowledge, this is the first study to clarify how antioxidant vitamins (A, D, and E) and trace elements (Cu, Mn, Se, and Zn) affect the metabolic, antioxidant and immunological gene expression profiles in dromedary camels throughout the transition phase. These effects were thoroughly studied in other livestock. Somagond et al. [37], for example, reported that repeated injections of multivitamins and multiminerals during the transition period enhance immune response by suppressing oxidative stress and inflammation in cows and their calves.

Blood neutrophils of the multiminerals and multivitamin groups showed higher mRNA expression of *GR-a, CD62L, CD11b, CD25*, and *CD44* and lower relative mRNA expression of *TLRs* and *CXCRs*. Furthermore, it was previously documented that endometrial expression of genes linked to antioxidant response was enhanced by supplementation with complex trace minerals (Zn, Mn, Cu, and Co) from day -30 to + 30 of parturition [38]. The impact of several dietary antioxidant supplements on the expression of genes and blood antioxidant indicators in young goats was investigated by [39]. The findings demonstrated that the expression of GPx mRNA and CuZn-SOD mRNA was up-regulated in the animals given either the Vit E/Se or Zn-Met diet.

It has been demonstrated that an animal's energy condition can affect the gene effect of metabolic gene [40]. According to [41], a model caused by a negative energy balance showed greater levels of expression for the metabolic genes. As a result, the increased metabolic marker expression seen in the current study may be linked to the increased energy requirement to sustain late gestation, calving and early lactation.

Cows experience metabolic stress during the postpartum period when their energy needs exceed their intake, putting them in a state of negative energy balance [42]. According to [43], the constitutive expression of the *IGF-I* gene was significantly greater one week postpartum and reached its highest value three weeks after calving. vitamin E (Vit E), selenium (Se), and zinc (Zn) are common antioxidants that are typically included in animal diets and improve various immune functions and health conditions. It is possible that the marked alteration in the expression profile of antioxidant markers as a result of vitamin A, D, E, and trace element supplementation (Cu, Mn, Se, Zn) [44].

The serum profile and gene expression of particular antioxidant indicators in dromedary camels had been investigated during the periparturient phase [10]. When compared to their values at calving, the *SOD1, SOD3, CAT, PRDX2, PRDX3, PRDX4, PRDX6, GPX*, and *AhpC/TSA* markers revealed up-regulations at (− 14) and (+ 14). In the current investigation, compared to the control group, the treated group had a lower expression profile of immune markers, indicating a lower level of stress and inflammatory response as well as less signals to the bone marrow to release immune cells [37]. Research on dairy cattle has demonstrated that multivitamins affect the phagocytic activity of neutrophils during stressful times as well as the proliferation, differentiation, and function of lymphocytes [45–47].

In addition, [48] found that the periparturient dairy cows' peripheral blood mononuclear cells (PBMC) expressed *IL-10* at its maximum on day 21 postpartum and at its lowest on the day of calving. The authors attributed this to the increased production of pro-inflammatory cytokines, such as IL-1b, IL-6, and TNF-a. The preceding papers demonstrate the potential reasons for changes in immunological marker expression profiles brought on by vitamin (A, D, and E) and trace element (Cu, Mn, Se, and Zn) supplementation.

No difference in glucose levels was seen between the examined animals, but higher levels of this metabolite were seen on the day of parturition in both treatment and control groups. This may have been caused by an increase in the stress hormone cortisol, necessary for gluconeogenesis to meet the sudden increase in demand for a rapid energy supply for the act of parturition and the start of lactation. The significant drain of glucose for lactose synthesis that occurs during the first few weeks of nursing after delivery may be the cause of the hypoglycemia [49]. This alteration may be related to specific trace minerals administered and their function in body metabolism as cofactors or catalysts in enzyme processes. These results were comparable to those previously reported in buffaloes [50] and in cows [51], but away from those observed in earlier research [52], which indicated constant mean glucose values around calving and postpartum time in Egyptian buffalo and Baladi cows.

While there was no change in cholesterol levels across treatments, both treatment and control groups had decreased levels of this metabolite on the day of parturition. The results of the current study were comparable to those from [51, 53]. The onset of ovarian activity and the formation of postpartum ovarian cyclicity, as well as the fact that cholesterol serves as a fatty acid transporter in the form of cholesterol ester, may all be related to the observed tendency of rising serum total cholesterol after calving for milk production.

Blood HDL and triglyceride levels were significantly affected by time (*P* < 0.001). At 21 days postpartum, there was a significant difference in both groups’ levels of HDL, which had increased from the prepartum period and parturition day. This finding is consistent with those made by [49], who found low HDL levels in treated or untreated groups, both in the prepartum and postpartum. In addition, our findings are similar to those made by [54], who verified lower values of this lipoprotein in the prepartum due to the energy requirement necessary for fetus growth and preparation of the mammary gland for milk production. Triglycerides were high during the prepartum period but suddenly decreased on the day of parturition, remaining at lower levels in both groups after parturition. In earlier investigations, this biochemical variable's values exhibited a similar pattern of behavior [51]. In contrast to our study [55], confirmed that the use of an injectable solution of selenium, copper, zinc, and manganese in dairy cows in the transition period decreased the contents of triglycerides.

Micro-mineral and vitamin injections had no discernible effect on blood total protein, although both treatment and control groups had reduced levels of this metabolite on the day of delivery. The results reported here were consistent with those found by [50]. However, [56] reported nearly stable plasma total protein concentrations around parturition and the postpartum period, whereas [52] detected increasing plasma total protein around parturition.

NEFA readings did not differ across treatments, but increased levels of this metabolite were seen in both the treatment and control groups on the day of parturition. These results corroborated those that had previously been published by [47, 51]. Cows that received subcutaneous injections of trace minerals during the transition period in the study by [55] had lower NEFA concentrations. Omur et al. [49] observed higher NEFA values in the control group when they employed trace minerals and vitamins A, D, and E in cows during the transition period. They proposed that vitamins and trace minerals may have an impact on lipomobilization and negative energy balance.

Between the treatment and control groups, there was no difference in BHBA values. However, greater BHBA values were found in both groups during the third postpartum week (Table 2). Our results were consistent with those found by [57]. These results were at odds with what was demonstrated by [49]. The latter authors employed vitamins A, D, and E as well as trace minerals in cows throughout the transition period, and they noticed decreased BHBA in the treated group of cows compared to the control group. It is unclear how adding trace minerals and vitamins to a diet reduce the concentration of BHBA. According to these authors, elevated BHBA values are linked to oxidative stress, a condition that can be seen in animals with high body scores at parturition and is influenced by the animal's metabolic state.

Serum cortisol levels only varied by day, with greater values in both groups on the day of parturition. This hormone's behavior has been seen by other researchers as well [47, 51]. Cortisol regulates gluconeogenesis, and during this time, a high lactose synthesis ensures milk production, necessitating the release of cortisol. Animals' immune systems are compromised during the transition period by hormonal and metabolic changes [58]. In contrast to the animals from control group, the treatment camels in our study had high cortisol levels at parturition but had considerable drops over the third week of lactation. This fact might be directly connected to the consumption of vitamins A and E and trace minerals. It is impossible to say whether this association also involves cortisol, although these factors were able to alter the immunological response in treatment animals.

In addition, a decrease in IGF-1 values was seen on the parturition day in both groups. Ramos et al. [59] observed decreased IGF-1 concentrations postpartum. This causes the somatotropic-IGF-1 axis to dissociate, preventing the stimulation of IGF-1 production by hepatocytes [60], and starting the process of insulin resistance for lipid mobilization for milk production [61]. There are few studies linking IGF-1 and mineral supplementation in dairy cows, [51] did not observe any effect of treatment when using trace minerals on this variable. IGF-1 remained within the parameters considered physiological [62] at all experimental moments in both groups, despite the reduced values at parturition.

Reactive oxygen species (ROS) are produced when the body's antioxidant capacity is exceeded, which causes oxidative stress and the suppression and degradation of appropriate immune system function [63]. High levels of oxidative stress during pregnancy make dairy camels more susceptible to metabolic and infectious illnesses [64]. In the current study, it has shown that multivitamins and trace minerals supplementation has improved antioxidant status via increase of GPx, SOD activity and TAC. This discovery was likely brought about by Zn, Cu, and Se's beneficial effects on controlling the body's balance of pro- and antioxidants through their participation in various antioxidant enzymes and enzymatic processes [65]. The Cu–Zn SOD, which is composed of copper and zinc, is what causes superoxide radicals to dismutate into hydrogen peroxide in the cytosol [66]. Due to its critical function in the removal of superoxide radicals generated during inflammation, Mn enhances the immunological response [67]. Zn plays an antioxidant role in addition to boosting cell replication and proliferation, which supports healthy innate and adaptive immunological responses [13]. According to a number of studies, vitamin and mineral administration improves immune function, fosters growth, and lowers the risk of infectious diseases by reducing perinatal oxidative stress[37]. Our findings diverged with those of [47], who claimed that injectable trace mineral supplementation in dairy cows had no impact on the serum concentration of GPx and SOD.

The availability of the vitamins A, C, E, and B6, as well as Fe, Zn, and Cu, has an impact on the inflammatory response, which connects innate and adaptive immunity [68]. Pro and anti-inflammatory cytokines are released simultaneously, according to previous research [69], and timely release of the anti-inflammatory cytokine is required for the regulation of the pro-inflammatory cytokines, which leads to the resolution of inflammatory disorders. Pro-inflammatory molecules like TNF-a, IL-1, IL6, and IFN-g are prevented from being produced by anti-inflammatory cytokines like IL-10, which reduces tissue damage and oxidative stress [70]. According to previous studies showing higher levels of pro-inflammatory cytokines in cows experiencing postpartum reproductive problems [71, 72], the significant increase in pro-inflammatory cytokines in control she camel may be due to a higher degree of calving stress and inflammatory condition as compared to treatment animals.

In comparison to groups who received micronutrient treatment, control cows may have lower levels of IL-10 as a result of an inflammatory immune response that is more active. Similar to our findings [48], showed that pro-inflammatory cytokines including IL-1, IL-6, and IL-10 were produced at higher rates in periparturient dairy cows on day 21 prepartum and reached their lowest levels on the day after calving due to higher manufacture of pro-inflammatory cytokines inclusive of IL-1β, IL-6 and TNF-a. Additionally [73], noted reduced concentrations of IL-10 in the milk and serum of cows who had subclinical mastitis. In comparison to the control group, the treatment group displayed the lowest levels of oxidative stress and inflammation as well as the strongest antioxidant capacity and immunological response. Additionally [37], found that the concentrations of IL-1α, IL-1β, IL6, IL8, and TNFα were significantly higher in the control group and significantly lower in the group that received multivitamin and mineral supplements. These inflammatory cytokines reached their highest levels on the day of calving and their lowest levels on day 21 after calving in all the groups. Furthermore, the IL-10 level was the highest on day 21 after calving and the lowest on the day of calving in all groups.

## Conclusion

Our results indicate that the combined injection of vitamins and minerals had the best impact on the animals' health status. A sufficient supply of these nutrients is essential for preserving a balanced immune response since they regulate the inflammatory response and reduce oxidative stress in periparturient she camels. Further research is needed to determine the right dosage and best combination of vitamins and trace elements based on the physiological stage, productivity, and health state of the animal in order to ensure maximum health and production without adverse effects.

## Data Availability

On reasonable request, the corresponding author will provide the information supporting the study's conclusions.

## Abbreviations

BHBA: Beta hydroxyl buterate
GPx: Glutathione peroxidase
HDL: High density lipoprotein
IGF-1: Insulin like growth factor 1
IL10: Interleukin 10
IL1-α: Interleukin 1 alpha
IL1-β: Interleukin 1 beta
IL6: Interleukin 6
NEFA: Non esterified fatty acid
SOD: Super oxide dismutase
TAC: Total antioxidant capacity
TNF-α: Tumor necrosis factor alpha
